# A therapeutic vascular conduit to support in vivo cell-secreted therapy

**DOI:** 10.1038/s41536-021-00150-2

**Published:** 2021-07-29

**Authors:** Edward X. Han, Hong Qian, Bo Jiang, Maria Figetakis, Natalia Kosyakova, George Tellides, Laura E. Niklason, William G. Chang

**Affiliations:** 1 Department of Biomedical Engineering, Yale School of Engineering and Applied Science, New Haven, CT USA; 2 Department of Anesthesiology, Yale School of Medicine, New Haven, CT USA; 3 Department of Surgery, Yale School of Medicine, New Haven, CT USA; 4 Department of Vascular Surgery, The First Hospital of China Medical University, Shenyang, China; 5 Department of Medicine, Section of Nephrology, Yale School of Medicine, New Haven, CT USA; 6 Veterans Affairs Connecticut Healthcare System, West Haven, CT USA; 7 Program in Vascular Biology and Therapeutics, Yale School of Medicine, New Haven, CT USA

**Keywords:** Tissue engineering, Anaemia, Cell delivery, Drug development, Hormonal therapies

## Abstract

A significant barrier to implementation of cell-based therapies is providing adequate vascularization to provide oxygen and nutrients. Here we describe an approach for cell transplantation termed the Therapeutic Vascular Conduit (TVC), which uses an acellular vessel as a scaffold for a hydrogel sheath containing cells designed to secrete a therapeutic protein. The TVC can be directly anastomosed as a vascular graft. Modeling supports the concept that the TVC allows oxygenated blood to flow in close proximity to the transplanted cells to prevent hypoxia. As a proof-of-principle study, we used erythropoietin (EPO) as a model therapeutic protein. If implanted as an arteriovenous vascular graft, such a construct could serve a dual role as an EPO delivery platform and hemodialysis access for patients with end-stage renal disease. When implanted into nude rats, TVCs containing EPO-secreting fibroblasts were able to increase serum EPO and hemoglobin levels for up to 4 weeks. However, constitutive EPO expression resulted in macrophage infiltration and luminal obstruction of the TVC, thus limiting longer-term efficacy. Follow-up in vitro studies support the hypothesis that EPO also functions to recruit macrophages. The TVC is a promising approach to cell-based therapeutic delivery that has the potential to overcome the oxygenation barrier to large-scale cellular implantation and could thus be used for a myriad of clinical disorders. However, a complete understanding of the biological effects of the selected therapeutic is absolutely essential.

## Introduction

Cell-based therapies involving implantation of living cells have tremendous potential for the treatment of a wide range of human illnesses, including genetic or acquired enzyme or hormone deficiencies. There are several key advantages of this approach over traditional medical treatments that intermittently dose small molecules or recombinant proteins. Cells have the potential to secrete continuously, can be regulated by drug-inducible promoters, and can even respond physiologically to changing patient conditions. Cell-based therapies have the advantage that the treated cells can be rigorously quality controlled and checked for safety prior to implantation into the patient. This is not possible in gene therapy approaches that rely upon viral vectors that have the potential for insertional mutagenesis, which can lead to unintended consequences, such as tumorigenesis^1,2^. In addition, injection of viral particles may lead to detrimental immune responses and liver toxicity^1,3^. Furthermore, if cell viability is adequately maintained, cell-based therapies can be robust and long-lasting.

Several different approaches for cell-based transplantation therapy have been investigated in recent years. Microencapsulation of protein-secreting cells in hydrogels (such as alginate) and implanting these microcapsules subcutaneously or intraperitoneally^4^ has been extensively studied. This has been used to investigate delivery of various proteins, such as insulin, factor IX, and hepatocyte growth factor^5–7^. Though studies utilizing microencapsulation show promise via in vitro and in vivo rodent studies, success in large mammals has remained elusive^8,9^. Fibrotic overgrowth, immune reaction, and hypoxia of the transplanted cells are key factors preventing clinical success of microencapsulated cells^10^. Larger microcapsule devices, such as tubular or planar cell chambers, are also being investigated for cell delivery but have similar issues with fibrosis and limited O_2_/nutrient diffusion leading to poor survival rates of the transplanted cells^10,11^. While cell-based therapies are promising, providing adequate oxygenation and nutrient delivery for cell survival is a critical hurdle that must be overcome^12^. Hypoxia and cell survival have proven to be a limiting factor preventing widespread clinical application of cell-based therapies^9,13^. As such, selection of an optimal site and tissue configuration for cell delivery are crucial considerations affecting the success of cell-based therapies.

To supply large quantities of cells with necessary O_2_ and nutrients, a vascular solution could be implemented^14^. To that end, we have designed the Therapeutic Vascular Conduit (TVC). The TVC utilizes a decellularized vessel as a starting scaffold. Genetically modified cells can then be situated immediately adjacent to the conduit using a hydrogel. The resultant TVC can then be directly anastomosed as a vascular graft that allows fully oxygenated arterial blood to flow through the vessel lumen, supplying O_2_ and nutrients to the cells that are coated on the outer surface.

We specifically tested secretion of a model hormone erythropoietin (EPO). Predominantly secreted by the interstitial cells of the kidney, EPO is upregulated in response to hypoxia to promote the survival, growth, and development of erythroid progenitors into red blood cells^15^. Anemia is one of the most common consequences of advanced chronic kidney disease and end-stage renal disease (ESRD), because renal disease leads to a relative deficiency of EPO that contributes to fatigue, reduced exercise tolerance, and negatively impacts patient quality of life. ESRD patients are routinely treated with recombinant forms of EPO to stimulate erythropoiesis^16^, but this is costly and potentially commits patients to life-long drug therapy.

Importantly, preparation of ESRD patients for hemodialysis includes surgical interventions to place a vascular catheter, an arteriovenous fistula, or an arteriovenous graft to facilitate access to the bloodstream for hemodialysis. Therefore, an EPO-secreting TVC (EPO-TVC) could theoretically serve a dual role as a platform for therapeutic hormone delivery and as a vascular access for hemodialysis. This article describes the theoretical and experimental work supporting the TVC design and demonstrates in vitro and in vivo efficacy of an EPO-TVC construct in a bioreactor system and an immunodeficient rat model.

## Results

### In silico modeling

Geometric assessment was initially performed to evaluate the benefits of the TVC compared to other three-dimensional (3D) cell transplantation structures. One major benefit of the TVC design is that the surface area-to-volume (SA:V) ratio remains constant when increasing the radius, as opposed to other 3D structures where the SA:V ratio decreases when increasing radius (Supplemental Fig. 1a). This point is further reinforced by the fact that, for an equivalent volume, the TVC allows for higher surface areas that can facilitate O_2_ and nutrient transfer (Supplemental Fig. 1b).

**Fig. 1:**
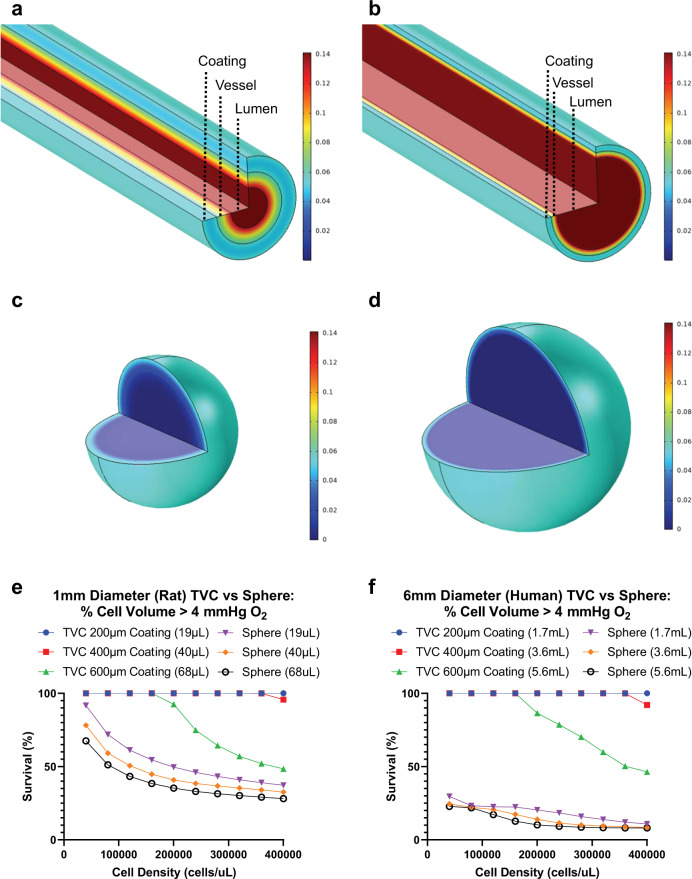
Therapeutic Vascular Conduit (TVC) in silico characterization. 3D models simulating diffusion of O_2_ in the TVC and O_2_ consumption by fibroblasts (FBs) seeded on the coating layer for **a** rat-sized and **b** human-sized TVCs. Equivalent volume spheres simulating consumption of O_2_ by FBs for **c** rat and **d** human-sized boluses. **e**, **f** Volume integration performed to determine percent coating volume above 4 mmHg (0.5%) O_2_, which correlates to predicted cell survival for varying cell densities.

We modeled O_2_ levels in a 3D reconstruction of the TVC. The in silico TVC had an inner lumen with flow, an acellular vessel in the middle layer, and an outer layer with a reaction module to simulate O_2_ consumption (Fig. 1a, b). Both rat- and human-sized TVCs were generated for analysis. Simulations were compared to an equivalent spherical volume of hydrogel containing an equivalent cell number—a sphere with the same volume and cell density as the sheath layer volume on a TVC (Fig. 1c, d). Cell densities tested in simulations ranged from 4.0 × 10^4^ to 4.0 × 10^5^ cells/μL, which allows for cell seeding numbers ranging from roughly 7.0 × 10^6^ to 2.2 × 10^9^ cells in a 6 mm diameter, 40 cm length human-sized TVC with 200–600 μm thick sheathes. This covers a wide range of potential cell transplant numbers. Final cell transplant number will be dependent upon secretion characteristics of genetically modified cells in the TVC.

**Fig. 2:**
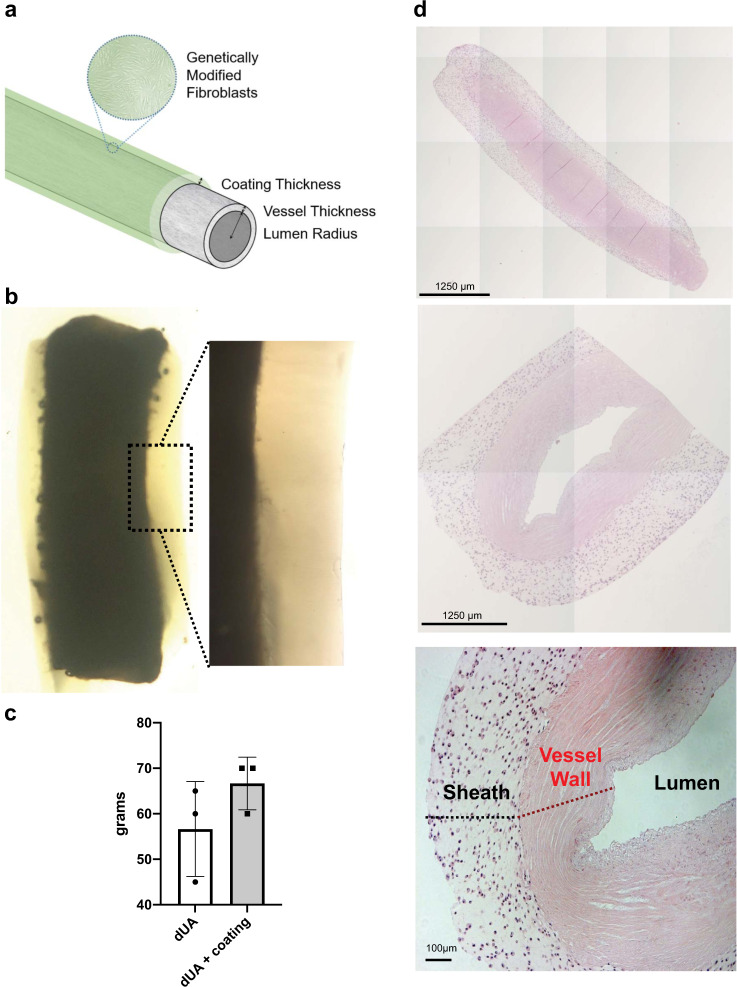
TVC design and creation. **a** Schematic representation of the TVC design with an acellular vessel and hydrogel coating. Inset illustrates that cultured, genetically modified FBs are incorporated into the coating. **b** Light microscopic gross/macroscopic view of an acellular vessel with a fibrin hydrogel coating. Hydrogel shown is cell free to illuminate the difference between the vessel and hydrogel coating. **c** Biomechanical property of suture retention strength was measured for dUA and coated dUA. Fibrin alone tested below the limits of assay (5 g). No statistically significant difference was observed between dUA and coated dUA (*p* = 0.2193). Means with standard deviations shown, *n* = 3 for each group. **d** Longitudinal and circular cross-sections of the TVC stained using H&E.

Cell survival in simulations was evaluated by determining the percent volume of cells above a critical O_2_ level of 4 mmHg (0.5% O_2_). Regions with O_2_ concentrations <4 mmHg were assumed to result in cell death due to anoxia^17^. Results indicated that the TVC design outperformed an equivalent volume spherical configuration in terms of cell survival (Fig. 1e, f). For example, a 1.5 cm length rat-sized TVC with 40 μL of a 400 μm sheath was predicted to have 100% cell survival up to a cell density of 3.6 × 10^5^ cells/μL (Fig. 1e). By comparison, a volume equivalent 40 μL sphere has only 35% survival at a density of 3.6 × 10^5^ cells/mL. The gap in predicted performance between TVCs and spherical volumes widens even further at human scale, where the TVC with a 400 μm sheath again has no cell death up to 3.6 × 10^5^ cells/μL while an equivalent spherical volume has only 9% survival at the same density (Fig. 2f).

Simulations show that sheath thickness plays a large role in the effectiveness of the TVC design. When the sheath thickness is 600 μm, negative effects on cell survival are observed at higher cell densities. Seeding cells at higher cell densities leads to diminishing returns for a 600 μm sheath thickness (Supplemental Fig. 2). Meanwhile, a low sheath thickness allows for high predicted cell survival percentages, since cells are in closer proximity to O_2_ available from the interstitial space and from the lumen. However, using a thin sheath also limits the total number of cells that can be seeded (Supplemental Fig. 2). As such, a sheath thickness of around 400 μm represents an optimal balance since it is thin enough to allow for diffusion and thick enough to support high numbers of total cells (Fig. 1e, f and Supplemental Fig. 2).

### TVC design and construction

The design of the TVC consists of genetically modified fibroblasts (FBs) situated within a hydrogel sheath surrounding an acellular vascular conduit (Fig. 2a). For rats, TVCs roughly 1–1.5 cm in length were constructed using decellularized human umbilical arteries (dUAs) coated with a fibrin sheath (Fig. 2b). Mechanical strength of the dUA was determined by suture retention and was not significantly altered by the fibrin coating (Fig. 2c). Pre-implantation histology reveals both the transverse and radial dimensions of the TVC are evenly coated with cells (Fig. 2d). The sheath layers were 400 μm in thickness while UA vessel walls were 400–500 μm in thickness.

### EPO-secreting cells

**Fig. 3:**
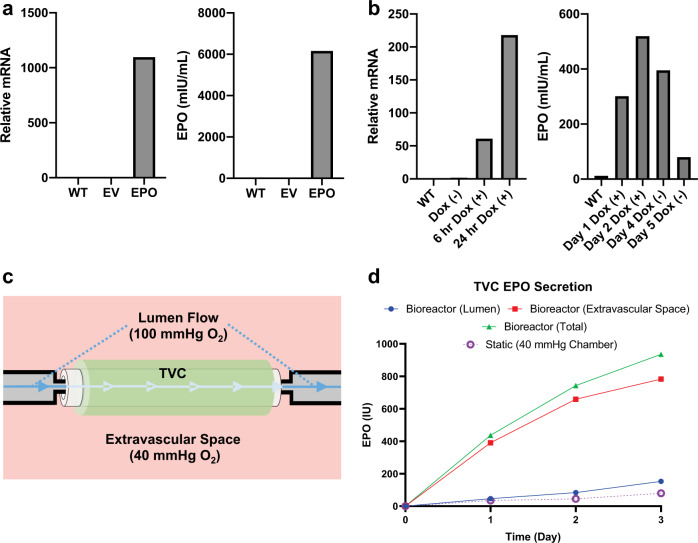
EPO secretion from genetically modified FBs and TVC testing. **a** *EPO* mRNA and EPO protein secretion from lentivirus-modified FBs programmed to constitutively secrete EPO. Wild-type (WT) and empty vector (EV) virus-infected cells were used as controls. **b** *EPO* mRNA and EPO protein secretion for FBs inserted with a doxycycline (dox)-inducible promoter for EPO secretion allowing for on/off signaling using dox. **c** Bioreactor set-up with the TVC with lumen flow through the center of the TVC. O_2_ in the lumen was maintained at 100 mmHg while the extravascular space was maintained at 40 mmHg. **d** Time course of EPO secretion from constitutive EPO-TVCs in the bioreactor setting and statically incubated at 40 mmHg O_2_. The majority of EPO was detected in the extravascular space of the bioreactor. For these experiments, *n* = 1.

To generate EPO-secreting FBs (EPO-FBs), human *EPO* was cloned into a lentivirus expression vector. The *EPO*-containing or empty vector control virus were used to infect human FBs under puromycin selection. The *EPO* mRNA expression in the EPO-FBs measured by real-time PCR was ~1000-fold greater than cells infected with the empty vector (Fig. 3a). At the protein level, 3.0 × 10^5^ EPO-FBs secreted 6 IU/mL of EPO after culture for 24 h in 500 μL of medium. There was no EPO detected from the empty vector-FBs (Fig. 3a). In order to titrate EPO secretion, a doxycycline (dox)-inducible promoter was used to regulate EPO expression in FBs. Dox-induced *EPO* mRNA expression was confirmed using real-time PCR (Fig. 3b), and induction of protein secretion was confirmed after 24 h of dox exposure (Fig. 3b).

### Bioreactor testing

In vitro bioreactor studies were performed to test EPO-TVC secretion capabilities. These bioreactors consist of a lumen reservoir containing medium pumped through the lumen of the TVC at a controlled rate 10 mL/min and set at 100 mmHg O_2_, and an extravascular space reservoir O_2_ set at 40 mmHg that supplied the volume surrounding the TVC (Fig. 3c). Medium was continually pumped through the lumen of the TVC and media samples for EPO measurements were taken at 24, 48, and 72 h. In all, 8.0 × 10^6^ FBs were incorporated into the sheath around the TVC and allowed for secretion of roughly 200–400 IU of EPO per day (Fig. 3d). Given that anemic ESRD patients receive 50–500 IU EPO/kg/week^18^, we anticipate that 2–20 IU of EPO daily should be more than adequate to stimulate an increase in the hemoglobin (Hgb) of a rat weighing 0.3 kg. Secretion of EPO from the TVC also demonstrated burst release secretion with a higher quantity of EPO after 1 day compared to afterwards. In the bioreactor, 90% of EPO was secreted into the extravascular space as opposed to 10% being secreted into the lumen. FBs are in closer contact with the extravascular space, while EPO must diffuse through the decellularized vessel in order to reach the lumen.

TVCs in the bioreactor set-up for 3 days were compared to TVCs statically incubated without flow in a hypoxia chamber at 40 mmHg O_2_ for 3 days. Results indicated that these static TVCs secreted less EPO than their counterparts in perfused bioreactors (Fig. 3d). This is likely because the bioreactor with 100 mmHg of O_2_ in the lumen facilitates higher O_2_ levels within the TVC and thus improved secretion characteristics.

### In vivo implantation of EPO-TVC

**Fig. 4:**
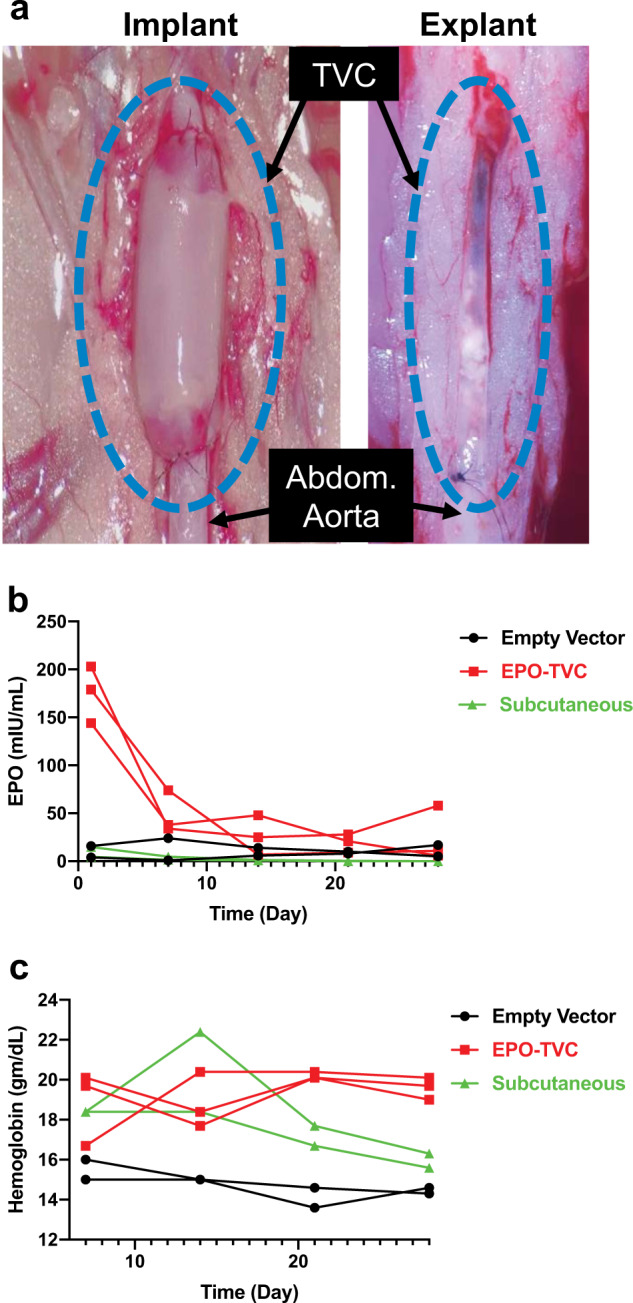
TVCs implanted into nude rats. **a** TVC implanted into rat as an end-to-end abdominal aorta graft and demonstrates integration and microvascularization when explanting at 3 months. **b** EPO secreting TVCs increase blood plasma EPO levels compared to empty vector TVCs and subcutaneous implants of EPO-FBs. **c** Hemoglobin (Hgb) levels were elevated for up to 1 month after implanting EPO-TVCs. From statistical analyses, EPO levels for empty vector control-TVC vs EPO-TVC *p* < 0.0001, EPO-TVC vs subcutaneous *p* < 0.0001, and empty vector control-TVC vs subcutaneous was not statistically different. Hgb levels control vs EPO *p* < 0.001, EPO vs subcutaneous *p* = 0.0384, and control vs subcutaneous *p* = 0.0493.

After confirming therapeutic levels of EPO secretion in vitro, we next determined whether the EPO-TVCs were capable of eliciting a sustained physiological effect in vivo. Nude rats were implanted with TVCs as end-to-end grafts in the abdominal aorta. This directly connected the TVC with the vascular system of the recipient rat and allowed for blood flow through the lumen (Fig. 4a). It should be noted that EPO-FBs secreted human EPO, which has been shown to be effective at stimulating the rat EPO receptor^19^ and increase red cell volume in rats^20^. As a control, subcutaneous implantation of fibrin hydrogels containing EPO-FBs, of the same volume and cell numbers, were implanted into separate animals. As another control, we performed implantations of TVCs with sheaths containing FBs infected with empty vector lentivirus.

Implants were maintained in vivo for up to 4 months. Explants taken at 2-week, 1-, 3-, and 4-month time points showed that TVCs were able to integrate with the host (Fig. 4a). Initial results showed a short-term spike in blood plasma EPO from animals implanted with EPO-TVCs (Fig. 4b). Afterwards, long-term EPO secretion was sustained at around 20 mIU/mL for the next 4 weeks. In contrast, only low levels of EPO were detected in the bloodstream of rats receiving the empty vector-TVC. For subcutaneous implants, again there was a burst release of EPO (up to 15 mIU/mL on 1 day post implant) followed by a continued decline, with minimal EPO detected after 7 days.

Hgb was also used as a metric to determine graft functionality. Rats implanted with EPO-TVCs had elevated Hgb levels, increasing from 15 to 20 g/dL after EPO-TVC transplantation (Fig. 4c). For the controls, while Hgb values for empty vector-TVC recipients remained unchanged, rats that received the EPO subcutaneous implants demonstrated elevated Hgb levels that peaked at around 2 weeks, before declining to normal levels by week 4. The initial burst of EPO was likely sufficient to induce a higher Hgb level in the rats with the subcutaneous implant. Because red cell mass is determined by both production and destruction, the persistently elevated Hgb in the absence of EPO elevation may have been due to a relative lack of red cell destruction. Histologically, no implanted human cells, as determined by anti-human leukocyte antigen (HLA) staining were detected at the 1-month time point for the subcutaneous implants (Supplemental Fig. 3).

**Fig. 5:**
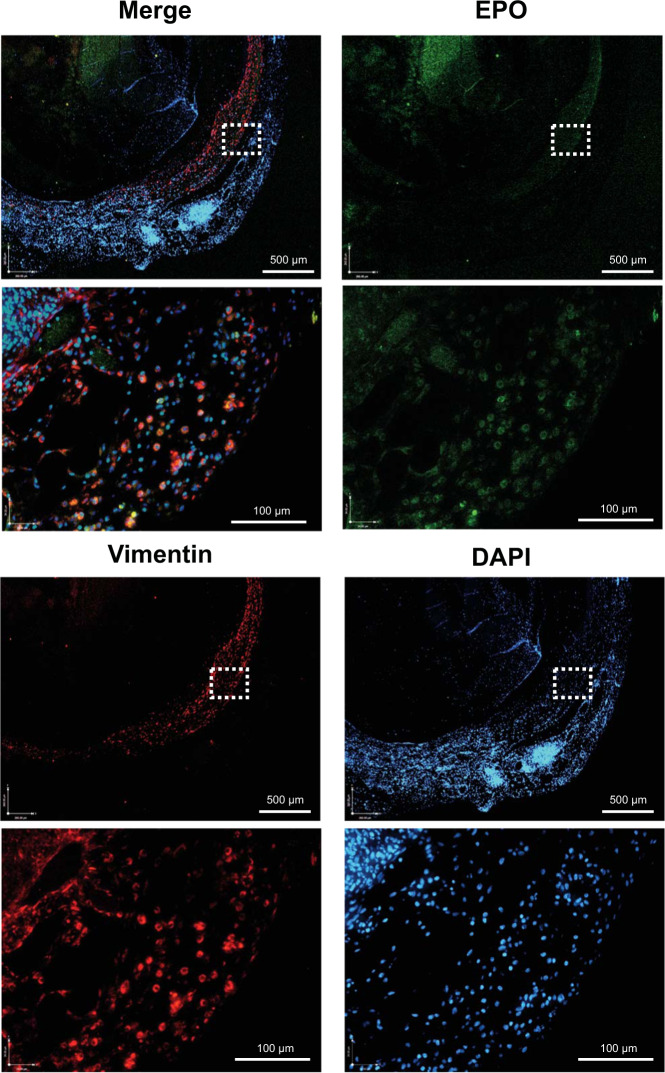
Immunofluorescent staining of EPO-TVC explant. TVC explant at 2 weeks stained with anti-EPO and anti-vimentin (FB marker) antibodies. Nuclei are stained with DAPI. TVC explant shows the outer layer of the TVC with EPO^+^ FBs.

**Fig. 6:**
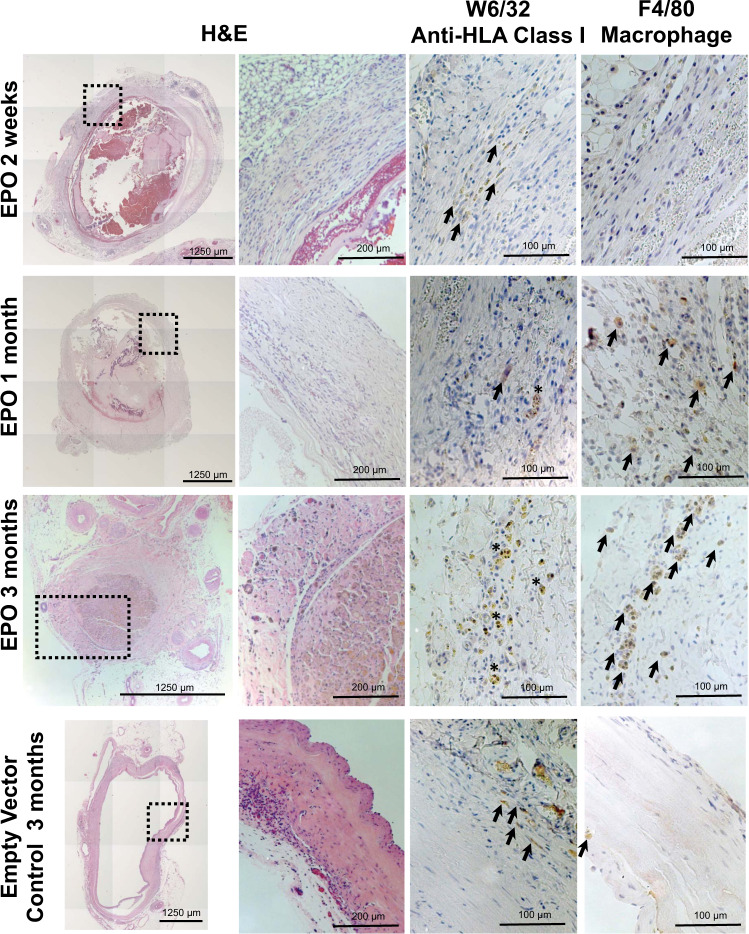
Immunohistochemistry staining of TVC explants. H&E staining shows the progression of luminal obstruction over the course of 3 months. W6/32 anti-HLA class I stains for human cells and highlights the modified human FBs implanted with the TVC. F4/80 macrophage staining is not present in the 2-week explant but becomes more pronounced in the 1- and 3-month explants. W6/32 with F4/80 at the 1- and 3-month time points suggests that macrophages were recruited and engulfed the transplanted EPO-FBs. In contrast, the empty vector control lumen remained patent for up to 3 months and had minimal F4/80 presence. Arrows highlight HLA^+^ human cells and F4/80^+^ macrophages and asterisks indicate phagocytic macrophages.

**Fig. 7:**
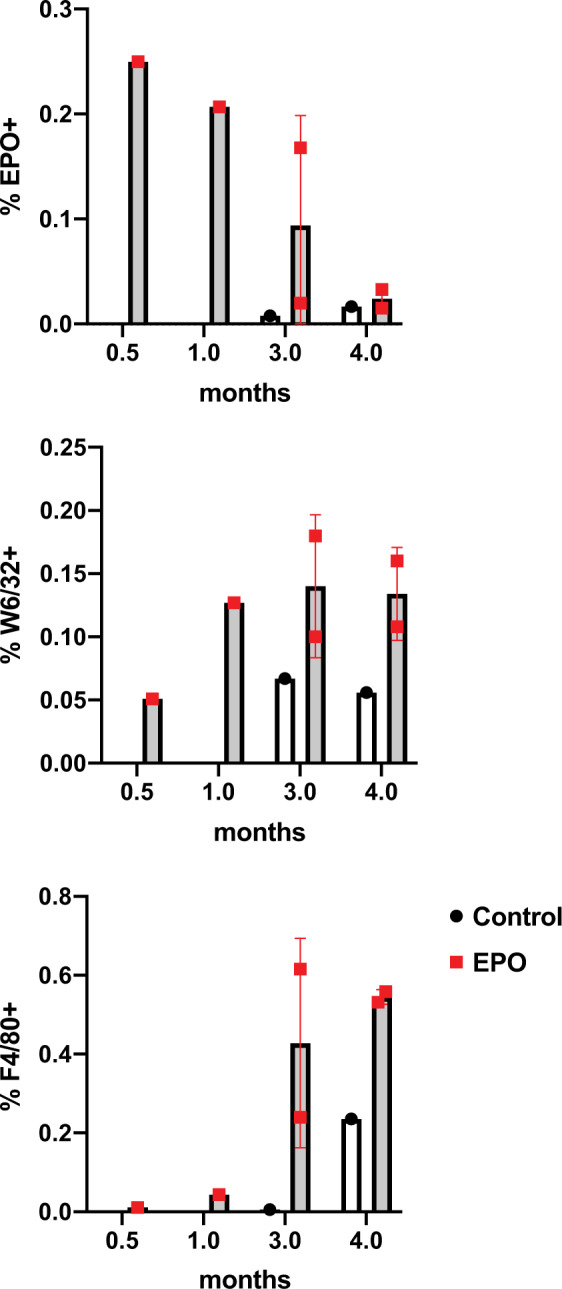
EPO, W6/32, and F4/80 staining of TVC explants. Implants from control and EPO-TVCs (2 weeks to 4 months) were stained for EPO by immunofluorescence and W6/32 (human HLA Class I) and F4/80 (macrophage marker) by immunohistochemistry. Control grafts were only examined at 3- and 4-month time points. Means with standard deviations are indicated for EPO 3- and 4-month time points where *n* = 2. For other time points, *n* = 1.

Staining of TVC explants for EPO and HLA at the 2-week through 4-month time points confirmed the survival of EPO-FBs (Figs. 5–7). EPO^+^ cells declined over the time course and appeared to localize to the periphery of the grafts. However, at the later time points of 3 and 4 months, we noted the consistent presence of significant vascular anomalies, including luminal occlusions (3 out of 4) or a pseudoaneurysm (1 out of 4), and macrophage infiltration in EPO-TVCs (Figs. 6 and 7 and Supplemental Fig. 4). Sustained EPO secretion, causing higher Hgb and blood viscosity, could contribute to increased risk of thrombosis in TVC conduits. However, intraluminal occlusions contained abundant macrophages as assessed by F4/80 immunostaining (Figs. 6 and 7). To confirm F4/80 staining of macrophages, an occluded 3-month implant was also stained with another macrophage marker, CD68 (Supplemental Fig. 5). In addition, these cells often co-stained with punctate intracellular HLA staining suggesting that the rat macrophages may have phagocytosed the human cells in the TVC. W6/32 staining for human cells therefore did not appear to show the same declining trend over the time course (Fig. 7). Image grafts analyzed can be viewed at 10.17632/sdhngz4ntn.2.

There are reports that EPO signaling promotes macrophage chemotaxis for the clearance of dead cells^21,22^. Hence, we speculated that EPO-secreting conduits may have stimulated the recruitment of host macrophages to the vessel lumen, leading to infiltration, occlusion, and to graft failure due to low blood flow and hypoxia. To test whether EPO secreted from FBs could recruit rat macrophages, a transwell study was performed to observe macrophage chemotaxis toward EPO-FBs (Fig. 7a). Significantly higher numbers of macrophages were observed to migrate through 8 μm pore transwells toward EPO-FBs as compared to empty vector control-FBs (Fig. 7b). This indicates that macrophage migration may have had an effect on EPO-TVC grafts.

### Regulated EPO-TVC

**Fig. 8:**
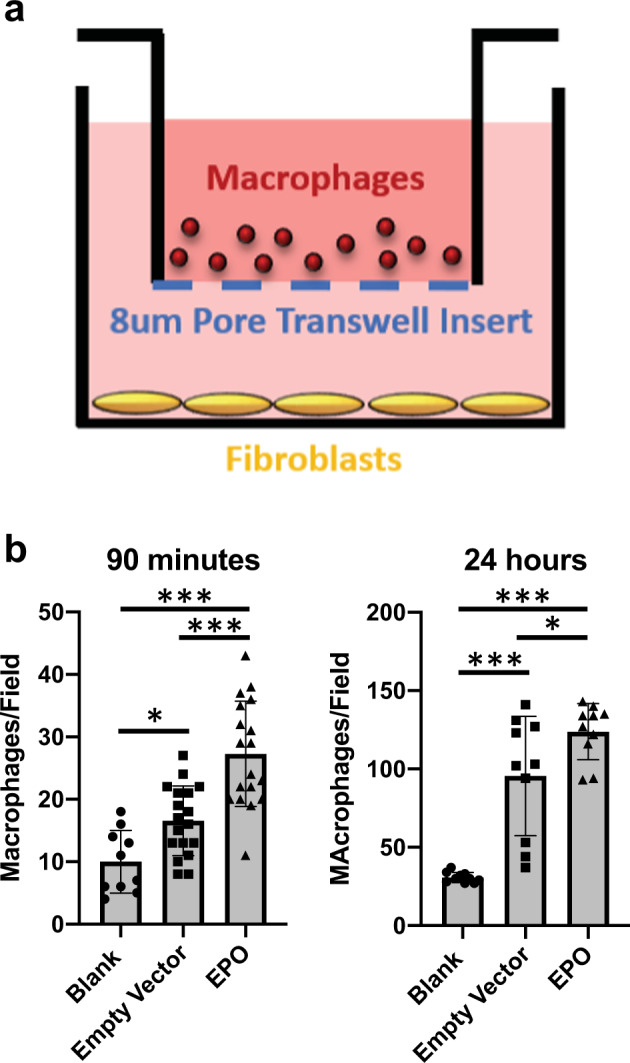
Macrophages migrate to EPO-FBs in vitro. **a** Set-up for macrophage migration assay. **b** Cell counts per field for wells after macrophages migrated through 8 μm pore transwells for 90 min or 24 h. From statistical analyses, at 90 min, blank vs empty vector-FBs *p* = 0.0470, blank vs EPO-FBs *p* < 0.0001, and empty vector-FBs vs EPO-FBs *p* < 0.0001. At 24 h, blank vs empty vector-FBs *p* < 0.0001, blank vs EPO-FBs *p* < 0.0001, and empty vector-FBs vs EPO-FBs *p* = 0.0387. *p < 0.05 and ***p < 0.0001. Means with standard deviations are indicated. For 90 min blank and 24 h time points, *n* = 10. For other conditions, *n* = 18.

To determine whether EPO secretion could be regulated, a dox-inducible EPO-TVC was also constructed and implanted in a nude rat as an abdominal aorta graft as previously described. When dox was administered via the drinking water, increases in plasma EPO concentration and Hgb were noted (Fig. 8).

## Discussion

Transplantation of therapeutic cells offers a potential long-term solution for patients suffering from protein or hormone deficiencies. However, transplantation of large quantities of cells has been limited by hypoxia that prevents adequate cell survival. Commonly utilized techniques such as subcutaneous and intraperitoneal transplantation rely solely on diffusive transport, limiting implant sizes to <200 μm without adequate vascularization^23^. Here we have described a new tissue engineering approach, the TVC, which incorporates a decellularized large vessel conduit that facilitates oxygenation of the therapeutic cells immediately after anastomosis to the host vasculature. Modeling results showed that the TVC is capable of supporting large densities of transplanted cells with a high degree of cell survival.

To test the TVC experimentally, we have chosen to first explore the therapeutic delivery of EPO. Because EPO is primarily secreted by the kidneys to maintain blood levels, this hormone becomes deficient in ESRD patients. The global prevalence of ESRD exceeds 2.5 million patients^24^. The majority of these patients require regular bolus dosing of costly recombinant EPO to maintain blood levels and prevent severe anemia^25^. A long-lasting implantable cellular source of EPO could significantly benefit ESRD patients, especially if a vascular graft is already needed for hemodialysis access.

**Fig. 9:**
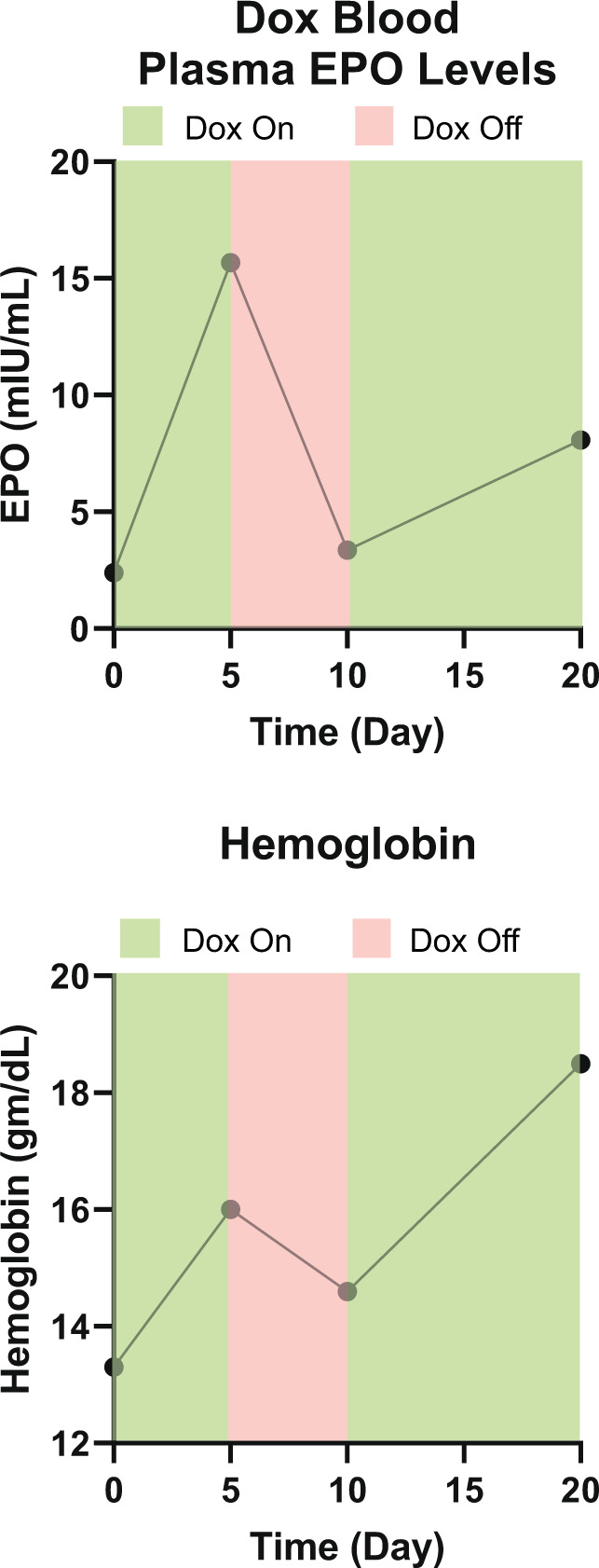
Regulated expression of EPO by dox-inducible promoter. In vivo regulation of EPO secretion with dox on/off time periods.

In this proof-of-concept study, we have demonstrated that EPO-secreting cells in the TVC are more effective at EPO delivery than subcutaneous implantation of cells. This supports our hypothesis that the co-implantation of an immediate vascular supply improves therapeutic efficacy. We have also demonstrated that the therapy can be regulated through a dox-inducible promoter (Fig. 9). Future iterations of the EPO-TVC could incorporate a hypoxia-inducible promoter to create a closed loop system, allowing cells to sense when increased EPO production is needed.

An unexpected finding in our study was that long-term constitutive expression of EPO led to cellular infiltration and occlusion of the TVC. Histological analyses and an in vitro macrophage migration assay indicated that this could be driven by macrophage infiltration. Indeed, macrophages have been shown to express the EPO receptor^26^, and EPO has been demonstrated to affect macrophage motility^27^. Other studies have noted that EPO stimulated chemotaxis of macrophages as well^21,22^. We hypothesize that this macrophage infiltration limits the long-term sustainability of the EPO secretion. However, because total occlusions were noted after the decline in EPO secretion, we cannot simply attribute the decline of EPO secretion to lack of luminal flow. Other macrophage effects such as release of inflammatory cytokines, reactive oxygen species, and/or additional biological factors might also be contributing to limit the sustainability of EPO secretion. Future experiments are needed to further elucidate the biological mechanisms limiting the therapeutic efficacy of the TVC.

These findings serve as an important lesson for future TVC applications. Although we envision that the TVC can be useful in a variety of clinical disorders, including treatment of genetic enzyme deficiencies, careful analyses of the full spectrum of biological effects of the intended therapeutic are essential. Special attention must also be paid to the effects on the vascular conduit of the TVC as well.

Looking forward, in addition to therapeutic selection and analysis of the full range of biological effects of the therapy, there are other important issues that need to be addressed prior to clinical application of this technology. For example, mitigation of the immune response to the transplanted tissue is a major consideration. Both fibrin and decellularized grafts are biocompatible components used in research and clinical settings. However, TVCs have not been approved by the Food and Drug Administration for purposes described here, and experiments shown represent investigational work only.

Ideally, immune suppression would not be necessary for this therapeutic approach. Eventual use of “universal donor cells,” such as immune-evasive major histocompatibility complex (MHC) class I/MHC class II knockout cells, might pave the way toward an allogeneic therapeutic product^28,29^. These cells could potentially be cryopreserved for future use. Another approach would be to use autologous cells from the recipient (such as dermal FBs or inducible pluripotent stem cells) that could be engineered to secrete the required protein and then re-transplanted within a TVC. Furthermore, genetic modification of cells carries with it the danger of introducing oncogenic properties that would be particularly detrimental for a long-term therapeutic. Gene targeting to genomic safe harbor locations^30^ may be a potential solution.

Overall, this study demonstrated that the TVC can be used to sustain cells that are genetically modified for specific protein secretion. Transplantation of the TVC can be clinically accomplished as an arteriovenous graft or arterial graft. This could be a useful means of delivering therapeutic quantities of cells into close proximity of the patient’s circulation and potential applications of this technology are wide-ranging.

## Methods

### In silico modeling in COMSOL Multiphysics

Computational modeling using finite element analysis in COMSOL Multiphysics® (Version 5.4-5.5 COMSOL) was performed to determine the O_2_ diffusion characteristics in the TVC design. A 3D model of the TVC design was created at both a rat-size scale—with a vessel radius of 0.5 mm and length of 15 mm—and at a human-size scale—with a vessel radius of 3 mm and length of 40 cm. Starting from the outside, the layers of the theoretical model represent the interstitial space, coating containing cells, decellularized vessel, and lumen. The Transport of Diluted Species Module in COMSOL was used to simulate the diffusion of O_2_ in the TVC. This module is governed by Fick’s First Law, defined as: (*J*_o2_ = *D*_o2_∇*C*_o2_), where *J*_o2_ is the molar flux of O_2_ [mol/(m^2^s)], *D*_o2_ is the diffusion coefficient of O_2_ [m^2^/s], and *C*_o2_ is the concentration of O_2_ [mol/m^3^]. The transport module was combined with the COMSOL laminar flow module to simulate the flow of blood/culture media through the lumen of the conduit. Laminar flow was governed by the Navier–Stokes and continuity equations assuming an incompressible fluid. For the fixed boundary conditions, the O_2_ level in the inlet of the conduit lumen was set to 100 mmHg to mirror fully oxygenated arterial blood flowing through the graft lumen. The interstitial space surrounding the outside of the coated conduit was set to 40 mmHg^31^. Inlet flow velocity through the lumen of the graft was set to 27 cm/s for rat TVCs^32^ and 0.9 m/s for human TVCs^33^. The O_2_ diffusion coefficients for decellularized vessels and fibrin were 1.25 × 10^−9^ m^2^/s^34^ and 1.7 × 10^−9^ m^2^/s^35^ respectively. Blood properties were defined with a density of 1050 kg/m^3^ and dynamic viscosity of 0.035 Pa·s^36^.

Determining survival of cells in the TVC configuration also required an equation to describe cellular O_2_ consumption of the coated cells. Importantly, since the wall of the vascular conduit is acellular, there is no net O_2_ consumption of the conduit wall but rather only from the cells that are coated on the outer surface. To model survival of cells, the Michaelis–Menten equation^34,37,38^: *R* = −*R*_max_ (*C*_o2_/*C*_o2_ + *K*_m_), was used to describe O_2_ consumption, where *R*_max_ is the maximal O_2_ consumption rate per unit volume [mol/(m^3^s)], *C*_o2_ is the concentration of O_2_ [mol/m^3^], and *K*_m_ is the half maximal concentration [mol/m^3^] that describes the *C*_o2_ needed to make *R* equal to half of *R*_max_. The literature value for maximum O_2_ consumption per cell was taken as 1.19 × 10^−17^ mol/(cell*s)^39^. This value can be multiplied by the volumetric cell density to convert O_2_ consumption per cell to O_2_ consumption per unit volume of coating on the conduit where *K*_m_ was set to 5.6 mmHg^39^. Simulations were meshed using COMSOL’s default “fine” element size and solved using the stationary solver. Simulation results computed O_2_ concentrations throughout the graft.

### Cloning and cell infection

Human *EPO* full-length cDNA (Origene, Rockville, MD, USA) was amplified by PCR using two cloning site-tagged primers and cloned into lentiviral vector pLenti-III-EF1α (abm, Richmond, BC, Canada) and dox-inducible lentiviral vector pCW57-MCS1-2A-MCS2 (a gift from Adam Karpf^40^ distributed by Addgene, Watertown, MA, USA). The inserts were verified by sequencing. Lentiviruses were produced by transfection of the lentiviral plasmid with packaging plasmids into 293T cells using Lipofectamine 2000. Human dermal FBs (ATCC®PCS-201-012, Manassas, VA, USA) were infected with lentiviruses for 16 h and then replaced with growth medium (Dulbecco’s Modified Eagle’s Medium (DMEM) + 10% fetal bovine serum (FBS) + 1× penicillin/streptomycin). After 48 h, the infected cells were selected and maintained in growth medium with 0.5 μg/mL puromycin to obtain the stable lines. *EPO* Tet-On FBs were induced by adding dox at 1 μg/mL in growth medium.

### mRNA quantification

Total RNA was isolated from human FBs using a RNeasy Mini Kit from QIAGEN (Germantown, MD, USA). Reverse transcription was performed using an Omniscript Kit (QIAGEN). For real-time PCR, 25 μL reactions contained 12.5 μL of 2× SYBR Green Supermix (Bio-Rad, Hercules, CA, USA), 0.4 μM of each primer, and 1 μL of template cDNA. Reactions were carried out in a C1000 Touch Thermal Cycler (Bio-Rad). Gene expression was calculated using the ΔΔCt method after normalization to glyceraldehyde 3-phosphate dehydrogenase (GAPDH) and reported as fold change. Primers for EPO were F 5’-AGGCCCTGTTGGTCAACTCT-3’ and R 5’-GCAGTGATTGTTCGGAGTGGA-3’. For GAPDH, they were F 5’-CCACTCCTCCACCTTTGACG-3’ and R 5’-CATGAGGTCCACCACCCTGT-3’.

### Vessel decellularization

dUAs were used as the acellular graft conduit to create TVCs. Preparation of dUAs was performed as previously described^41^. Briefly, human umbilical cords, designated as discarded tissue, were obtained from the Yale New Haven Hospital. UAs were mechanically separated from the umbilical cord using forceps. Isolated arteries were placed into CHAPS buffer overnight. The following day, arteries were rinsed using phosphate-buffered saline (PBS) and placed into sodium dodecyl sulfate buffer overnight at 37 °C. Extensive washes with PBS were then performed to remove any residual detergents. The dUAs were then placed in 10% FBS/10% penicillin/streptomycin (P/S) PBS solution in order to remove residual DNA. The decellularized vessels were then stored at 4 °C in 1% P/S PBS.

### Engineered graft construction

TVCs were created using 10 mg/mL bovine fibrinogen prepared in DMEM medium with 1 mM CaCl_2_. To prepare for the molding procedure, a 4 mm inner diameter polypropylene mold was coated with 5% pluronic for 30 min and washed with PBS. To hold the dUA, a 14-gauge blunt metal syringe was cannulated through the lumen. FBs were suspended in fibrinogen and combined with thrombin at a 100 mg fibrinogen: 1 U thrombin ratio. Immediately after mixing and prior to gelation, the fibrin/cell mixture was then transferred into the polypropylene mold with the dUA and the assembly was incubated at 37 °C to allow the fibrin to polymerize for 20 min. The blunt metal syringe was then gently pulled away from the mold to isolate the dUA with the fibrin/cell coating. TVCs were then immediately used in either in vitro or in vivo experiments. Biomechanical strength as determined by suture retention was carried out as previously described^42^.

### EPO and Hgb quantification

In vitro testing of EPO secretion from genetically modified FBs was performed by seeding 3.0 × 10^5^ cells in 24-well culture plates. For constitutive EPO-secreting FBs, media was collected after 24 h and assayed using a DuoSet® Human Erythropoietin enzyme-linked immunosorbent assay (ELISA) Kit (R&D system, Minneapolis, MN, USA). For in vitro quantification of dox-regulated EPO secretion, dox was provided at 1 μg/mL for days 1–3 and removed on days 4–5. For in vivo regulation, 0.5 mg/mL dox was added to the rat drinking water.

Quantification of EPO secretion from implanted cells was determined after collection of rat blood by tail nick and centrifugation at 5000 × *g* for 10 min at 4 °C. EPO was quantified in the resulting rat plasma supernatant by using an EPO ELISA Kit. Tail vein nicks were also used to obtain blood samples for testing Hgb levels quantified with an i-Stat loaded with an EC8^+^ cartridge (Abbott Laboratories, Abbott Park, IL, USA). Each measurement reports is from a distinct blood sample.

### Bioreactors

Bioreactors housing TVC constructs were used to test the EPO secretion of FBs in TVCs. TVCs were first cannulated and mounted in the bioreactor set-up. The extravascular space surrounding the TVC was kept at 40 mmHg O_2_ while the medium flowing through the lumen of the TVC was kept at 100 mmHg O_2_. The pump was run at 10 mL/min to mimic average rat blood flow rates^43^. The bioreactor was run for up to 3 days and samples were collected daily to assess EPO secretion capabilities of the graft. As a control, TVCs were also statically incubated in a hypoxia chamber submerged in medium at 40 mmHg O_2_ for up to 3 days, with daily sampling to determine EPO secretion.

### Animals and TVC surgical implantation

Athymic male nude rats (NIH-*Foxn1*^*rnu*^) were purchased from Charles River Labs (Wilmington, MA, USA) for transplantation experiments. Rats were 6–8 weeks of age when used for surgery. Sprague Dawley rats (Charles River Labs) age 8–12 weeks were sacrificed for harvest of alveolar macrophages. All protocols involving these nude and Sprague Dawley rats were approved by Yale Institutional Animal Care and Use Committee, and studies were performed in accordance with the Animal Research: Reporting In Vivo Experiments guidelines and National Institutes of Health Guide for the Care and Use of Laboratory Animals. In this study, we grafted 6 animals with EPO-TVC implants (three of which had serial long-term EPO and Hgb measures carried out), 2 animals had control-TVC (with serial EPO and Hgb), 2 animals had subcutaneous implants (with serial EPO and Hgb), and one animal had a dox-inducible TVC implant. Animals were only excluded from the study if there was a technical failure of the surgical graft.

TVCs were implanted into nude rats as end-to-end interposition grafts at the abdominal aorta to test EPO secretion capabilities in vivo. Nude rats were anesthetized using isoflurane and injected with buprenorphine and bupivacaine for analgesia. The vascular surgeon was blinded to the study groups of the animals. A sterile field was prepared and a midline incision was made. The infrarenal abdominal aorta was then bluntly dissected away from the surrounding tissue and two microvascular clamps are used to stop blood flow temporarily. A transection was made between the two clamps and the TVC was anastomosed to the proximal and distal ends of the rat abdominal aorta transection. Clamps were removed and blood flow through the graft was visually confirmed. The intestines were then returned into the peritoneal cavity and the abdomen was closed.

### Immunohistochemistry and immunofluorescence

For histology, TVCs were fixed in 10% neutral-buffered formalin overnight. Samples were embedded in paraffin, sectioned, and stained with hematoxylin and eosin, W6/32 anti-HLA Class I (Cat. #311402, Biolegend, San Diego, CA, USA, dilution 1:100), or anti-F4/80 macrophage marker (Cat. #MA5-16363, Thermo Fisher Scientific, Waltham, MA, USA, dilution 1:75). Although better known as a murine macrophage marker, F4/80 is expressed by rat macrophages as well^44^. For further confirmation, we also stained with another macrophage marker CD68 (Cat# ABIN3030412, antibodies-online.com, dilution 1:100). For immunofluorescence, slides were baked at 65 °C and sequentially washed in xylene and 100, 95, 85, 75, and 50% EtOH. They were then placed in sodium citrate buffer at 70 °C for 20 min for antigen retrieval. Following a PBS wash, blocking buffer was applied for 1 h. Slides were then incubated overnight at 4 °C with primary antibody in blocking buffer. Primary antibodies used were EPO (Cat. #MA5-15684, Thermo Fisher Scientific, dilution 1:200) and vimentin (Cat. #SC-7557, Santa Cruz Biotechnology, Dallas, TX, USA). The following day, slides were incubated with secondary antibody at room temperature for an hour before washing to remove unattached secondary antibody. Fluorescent imaging was performed on a Leica DMI600B fluorescence microscope with the Leica Las X Life Science Software (Buffalo Grove, IL, USA).

To determine the percentage of cells that stained positively for EPO, W6/32, and F4/80 across a time course of graft implantation, >1000 cells were manually counted from randomly selected fields of view that incorporated the vessel wall and lumen of the graft. Images were scored in a blinded manner.

### Macrophage migration assay

Alveolar macrophages were obtained and cultured using a previously published protocol^45^. For the migration assay, 4.0 × 10^4^ constitutive EPO-FBs or empty vector-FBs were cultured in 12-well plates. Macrophages were incubated with Vybrant™ DiI membrane dye for 5 min at 37 °C and centrifuged/washed with DMEM media to remove excess dye. 8.0 μm transwells (Falcon #353182) were placed in the 12-well plates containing medium only, EPO-FBs, or empty vector-FBs. In all, 5.0 × 10^4^ macrophages were then placed into the top of the transwell. Quantification of macrophage migration across the transwell membrane was carried out at 90 min and 24 h using a Leica DMI600B fluorescence microscope.

### Statistical analysis

For comparison of EPO and Hgb values obtained for in vivo experiments, mixed-effects analyses were used. For the macrophage migration assay, one-way analysis of variance analyses were used. For suture retention test, an unpaired *t* test was performed. GraphPad Prism 9 software (San Diego, CA, USA) was used for statistical analyses.

### Ethics

Protocols were reviewed by the Yale Institutional Review Board (IRB) (ID 2000025795), and it was determined that experiments described are not considered human subject research and thus do not require IRB approval.

### Reporting summary

Further information on research design is available in the Nature Research Reporting Summary linked to this article.

## Supplementary information

Supplementary Information

Reporting Summary

## Data Availability

The raw data required to reproduce these findings are available to download from 10.17632/sdhngz4ntn.2.

## Code availability

COMSOL Multiphysics® Version 5.4-5.5 was used for mathematical modeling. GraphPad Prism 9 was used for statistical analyses.
