# Supplementary material for: A therapeutic vascular conduit to support in vivo cell-secreted therapy
Source: NPJ Regen Med. 2021 Jul 29;6:40. doi: 10.1038/s41536-021-00150-2 (PMC8322381; doi:10.1038/s41536-021-00150-2)
Supplement: Supplementary file 1 — Supplementary Information [file 41536_2021_150_MOESM1_ESM.pdf]

a

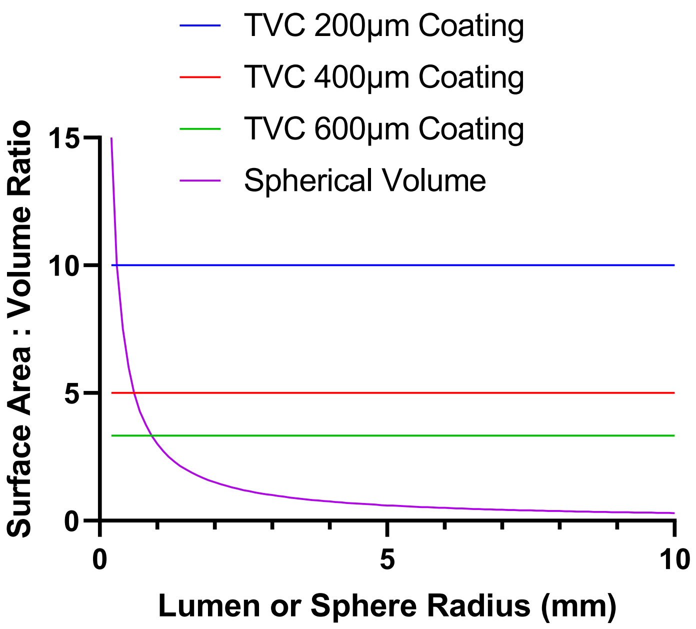

b

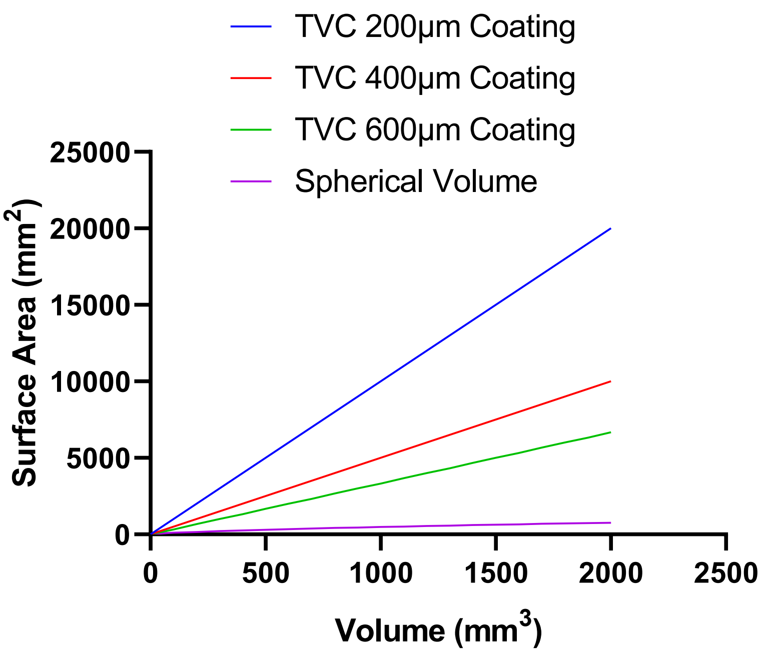

Supplementary Figure 1. Geometric benefits of TVC. (a) Surface area to volume ratio of TVCs of varying coating thicknesses versus a spherical volume. When increasing the radius of the TVC to make a scaled-up, human-sized tissue, the surface area to volume ratio remains constant. In contrast, increasing the radius of a spherical volume significantly decreases the surface area to volume ratio, which decreases O_2 and nutrient transfer available per unit volume. (b) For given volumes, the TVC is capable of supporting much higher surface areas than spherical volumes.

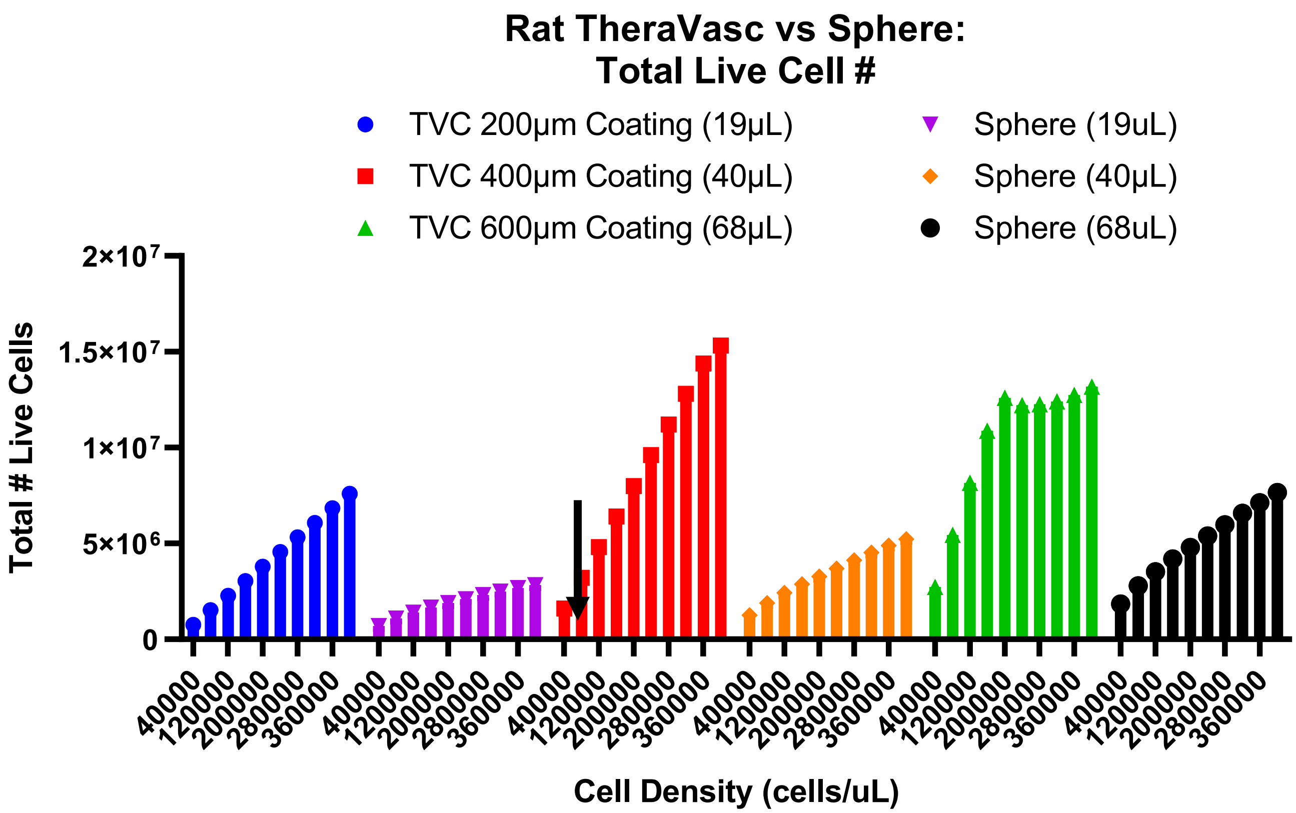

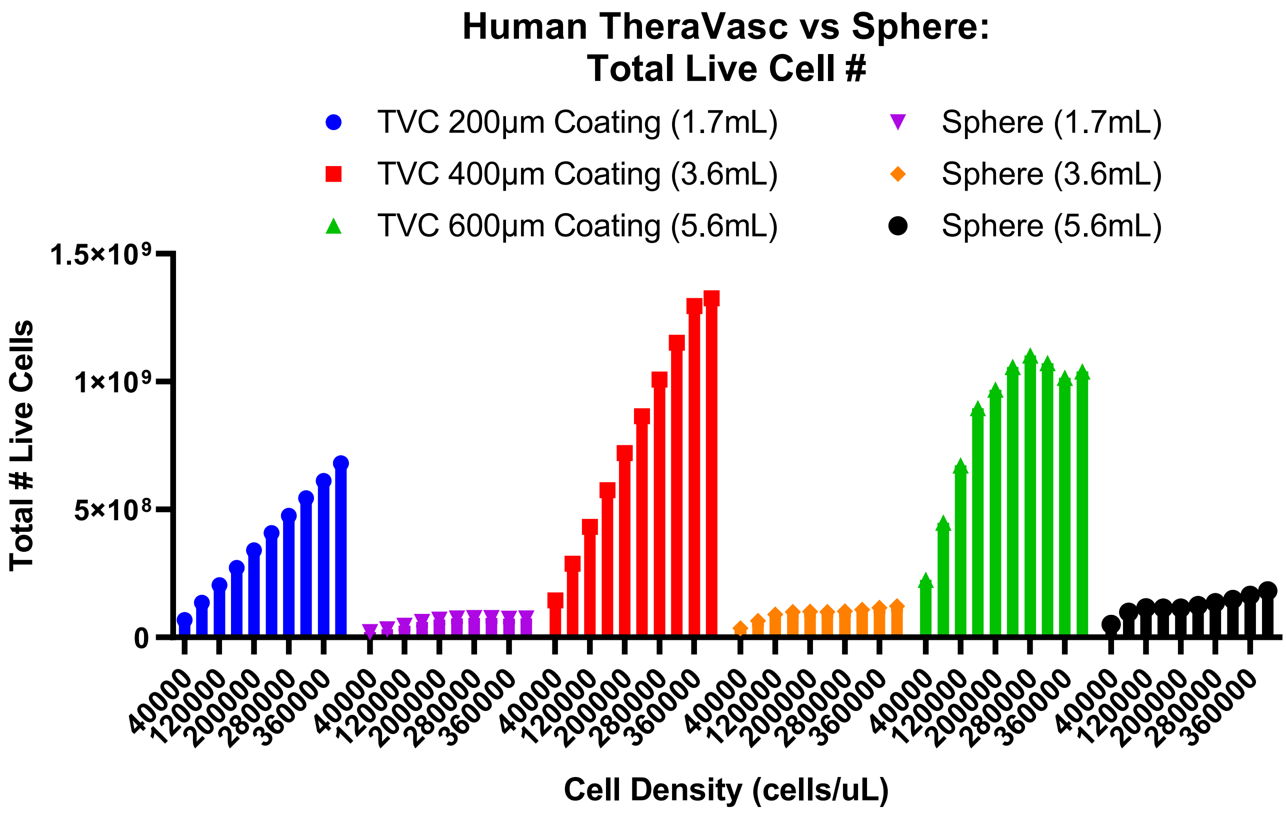

Supplementary Figure 2. Simulations for number of living cells in TVCs and spheres. Total number of live cells predicted by combining survival % data with cell density. TVCs at 200 µm have less coating volume and thus supports less cells while TVCs with a 600 µm coating have lower survival rates leading to diminishing returns for seeding at high cell densities. TVCs at 400 µm scale well when increasing cell density. Arrow indicates final cell density and coating thickness used for rat TVCs.

W6/32
Anti-HLA Class I

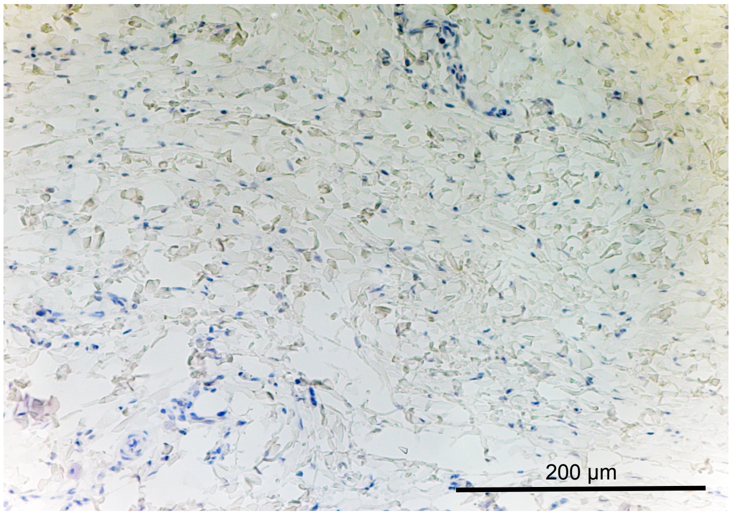

Supplementary Figure 3. Subcutaneous explant. W6/32 HLA staining of subcutaneous explants created using fibrin with EPO-FBs at an equivalent volume and cell density as rat EPO-TVCs. No positive cells were detected.

Identifier	Group	Duration	Vessel Patency
TVC1	EPO	2 weeks	open
TVC2	EPO	4 months	occlusion
TVC3	EPO	3 months	occlusion
TVC4	Control	3 months	open
TVC5	EPO	4 months	occlusion
TVC6	Control	4 months	open
TVC7	EPO	3 months	pseudoaneurysm
TVC8	EPO	1 month	open
TCV-dox	inducible-EPO	2 months	open

Supplementary Figure 4. Description of characteristics and patency of TVC Implants. A total of 9 TVCs were implanted for 2 weeks to 4 months. 6 EPO-, 2 control-, and 1 dox-inducible TVCs were implanted. All long-term EPO-TVCs (3 and 4 months) developed occlusions or in one case (TVC7) a pseudoaneurysm, while neither of the control-TVCs developed occlusions.

a

CD68

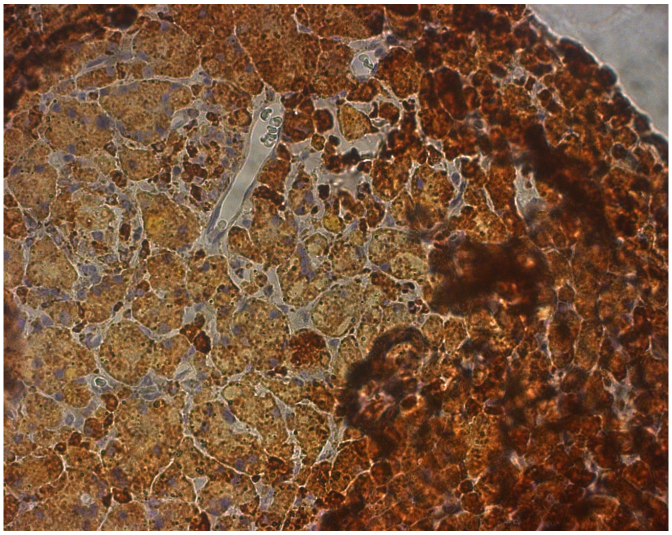

b

F4/80

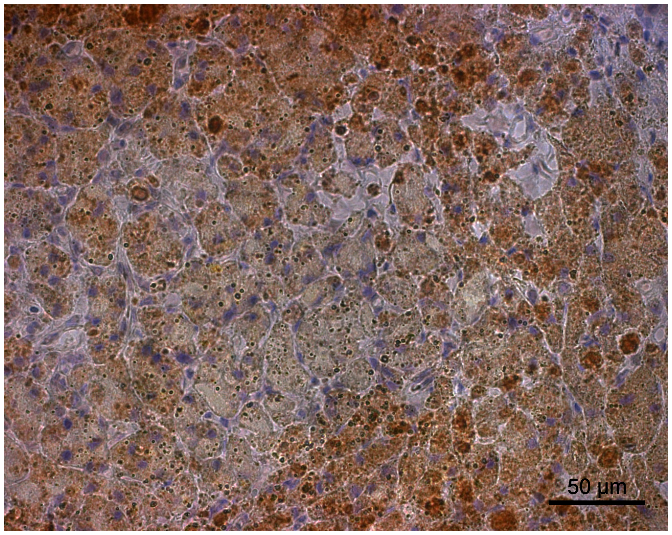

Supplementary Figure 5. Staining TVC occlusions with macrophage markers CD68 and F4/80. EPO-TVC after 3 months demonstrated macrophage rich occlusions stained with both (a) CD68 and (b) F4/80. Scale bar for both is 50 µm.
