# Supplementary material for: A therapeutic vascular conduit to support in vivo cell-secreted therapy
Source: NPJ Regen Med. 2021 Jul 29;6:40. doi: 10.1038/s41536-021-00150-2 (PMC8322381; doi:10.1038/s41536-021-00150-2)
Supplement: Supplementary file 2 — Reporting Summary [file 41536_2021_150_MOESM2_ESM.pdf]

nature research

Corresponding author(s): William G. Chang

Last updated by author(s): Jun 23, 2021

Reporting Summary

Nature Research wishes to improve the reproducibility of the work that we publish. This form provides structure for consistency and transparency in reporting. For further information on Nature Research policies, see our Editorial Policies and the Editorial Policy Checklist.

Statistics

For all statistical analyses, confirm that the following items are present in the figure legend, table legend, main text, or Methods section.

n/a	Confirmed	
☐	☒	The exact sample size (*n*) for each experimental group/condition, given as a discrete number and unit of measurement
☐	☒	A statement on whether measurements were taken from distinct samples or whether the same sample was measured repeatedly
☐	☒	The statistical test(s) used AND whether they are one- or two-sided<br>*Only common tests should be described solely by name; describe more complex techniques in the Methods section.*
☒	☐	A description of all covariates tested
☒	☐	A description of any assumptions or corrections, such as tests of normality and adjustment for multiple comparisons
☐	☒	A full description of the statistical parameters including central tendency (e.g. means) or other basic estimates (e.g. regression coefficient) AND variation (e.g. standard deviation) or associated estimates of uncertainty (e.g. confidence intervals)
☐	☒	For null hypothesis testing, the test statistic (e.g. *F*, *t*, *r*) with confidence intervals, effect sizes, degrees of freedom and *P* value noted<br>*Give P values as exact values whenever suitable.*
☒	☐	For Bayesian analysis, information on the choice of priors and Markov chain Monte Carlo settings
☒	☐	For hierarchical and complex designs, identification of the appropriate level for tests and full reporting of outcomes
☒	☐	Estimates of effect sizes (e.g. Cohen's *d*, Pearson's *r*), indicating how they were calculated

Our web collection on statistics for biologists contains articles on many of the points above.

Software and code

Policy information about availability of computer code

Data collection	COMSOL Multiphysics (Version 5.4-5.5) was used.
Data analysis	GraphPad Prism 9 was used.

For manuscripts utilizing custom algorithms or software that are central to the research but not yet described in published literature, software must be made available to editors and reviewers. We strongly encourage code deposition in a community repository (e.g. GitHub). See the Nature Research guidelines for submitting code & software for further information.

Data

Policy information about availability of data

All manuscripts must include a data availability statement. This statement should provide the following information, where applicable:

- Accession codes, unique identifiers, or web links for publicly available datasets
- A list of figures that have associated raw data
- A description of any restrictions on data availability

The raw data required to reproduce these findings are available to download from http://dx.doi.org/10.17632/sdhngz4ntn.2

Field-specific reporting

Please select the one below that is the best fit for your research. If you are not sure, read the appropriate sections before making your selection.

☒ Life sciences ☐ Behavioural & social sciences ☐ Ecological, evolutionary & environmental sciences

For a reference copy of the document with all sections, see nature.com/documents/nr-reporting-summary-flat.pdf

Life sciences study design

All studies must disclose on these points even when the disclosure is negative.

Sample size	No sample size calculation was performed
Data exclusions	Animals in which there were technical problems with the graft implantation were excluded
Replication	Individual data points and replicates are included in the manuscript and figures.
Randomization	The was systematic randomization performed.
Blinding	The surgeon implanting the grafts was blinded to the groups.

Reporting for specific materials, systems and methods

We require information from authors about some types of materials, experimental systems and methods used in many studies. Here, indicate whether each material, system or method listed is relevant to your study. If you are not sure if a list item applies to your research, read the appropriate section before selecting a response.

Materials & experimental systems

n/a	Involved in the study	
☐	☒	Antibodies
☐	☒	Eukaryotic cell lines
☒	☐	Palaeontology and archaeology
☐	☒	Animals and other organisms
☒	☐	Human research participants
☒	☐	Clinical data
☒	☐	Dual use research of concern

Methods

n/a	Involved in the study	
☒	☐	ChIP-seq
☒	☐	Flow cytometry
☒	☐	MRI-based neuroimaging

Antibodies

Antibodies used	anti-F4/80 macrophag marker (Thermo Fisher MA5-16363)<br>anti-CD68 (antibodies-online.com ABIN3030412)<br>W6/32 anti-human leukocyte antigen (Biolegend 311402)<br>EPO antibody (Thermo Fisher Ma5-15684)<br>vimentin antibody (Santa Cruz SC-7557)
Validation	For F4/80, https://www.thermofisher.com/antibody/product/F4-80-Antibody-clone-SP115-Monoclonal/MA5-16363 describes validation and references.<br>For anti-CD68, https://www.antibodies-online.com/antibody/3030412/anti-CD68+Molecule+CD68+AA+312-326+antibody/ describes validation and references.<br>For W6/3, https://www.biolegend.com/en-us/products/purified-anti-human-hla-a-b-c-antibody-1874?GroupID=GROUP28 describes validation and references.<br>For EPO, https://www.thermofisher.com/antibody/product/Epo-Antibody-clone-4F11-Monoclonal/MA5-15684 describes validation.<br>For vimentin, https://www.scbt.com/p/vimentin-antibody-c-20 describes validation and references.

Eukaryotic cell lines

Policy information about cell lines

Cell line source(s)	human dermal fibroblasts were obtained from the ATCC
Authentication	Cells display the appropriate morphology, adherence, and + vimentin staining.

Mycoplasma contamination	Cell lines are routinely tested for Mycoplasma.
Commonly misidentified lines (See ICLAC register)	*Name any commonly misidentified cell lines used in the study and provide a rationale for their use.*

Animals and other organisms

Policy information about studies involving animals; ARRIVE guidelines recommended for reporting animal research

Laboratory animals	Athymic nude rats NIH-Foxn1 RNU and Sprague Dawley from Charles River Labs, were male, and age 6-8 weeks at time of surgery.
Wild animals	*Provide details on animals observed in or captured in the field; report species, sex and age where possible. Describe how animals were caught and transported and what happened to captive animals after the study (if killed, explain why and describe method; if released, say where and when) OR state that the study did not involve wild animals.*
Field-collected samples	*For laboratory work with field-collected samples, describe all relevant parameters such as housing, maintenance, temperature, photoperiod and end-of-experiment protocol OR state that the study did not involve samples collected from the field.*
Ethics oversight	Animal experiments were approved by the Yale Instiutional Animal Care and Use Committee and studies were performed in accordance with ARRIVE guidelines. Protocols were reviewed by the Yale IRB, and it was determined that research was not considered human subjects research.

Note that full information on the approval of the study protocol must also be provided in the manuscript.
